# 3D Printed Polycaprolactone/Gelatin/Bacterial Cellulose/Hydroxyapatite Composite Scaffold for Bone Tissue Engineering

**DOI:** 10.3390/polym12091962

**Published:** 2020-08-29

**Authors:** Abdullah M. Cakmak, Semra Unal, Ali Sahin, Faik N. Oktar, Mustafa Sengor, Nazmi Ekren, Oguzhan Gunduz, Deepak M. Kalaskar

**Affiliations:** 1 Department of Bioengineering, Faculty of Engineering, Marmara University, 34722 Istanbul, Turkey; mukreminncakmak@gmail.com (A.M.C.); semra.unal@marmara.edu.tr (S.U.); foktar@marmara.edu.tr (F.N.O.); 2 Center for Nanotechnology & Biomaterials Application and Research (NBUAM), Marmara University, 34722 Istanbul, Turkey; mustafa.sengor@marmara.edu.tr (M.S.); nazmiekren@marmara.edu.tr (N.E.); 3 Institute of Neurological Sciences, Marmara University, 34722 Istanbul, Turkey; 4 Department of Biochemistry, School of Medicine/Genetic and Metabolic Diseases Research and Investigation Center, Marmara University, 34722 Istanbul, Turkey; alisahin@marmara.edu.tr; 5 Department of Metallurgy and Materials Engineering, Faculty of Technology, Marmara University, 34722 Istanbul, Turkey; 6 Department of Electrical and Electronics Engineering, Faculty of Technology, Marmara University, 34722 Istanbul, Turkey; 7 UCL Division of Surgery and Interventional Sciences, Royal Free Hospital Campus Rowland Hill Street, London NW3 2PF, UK

**Keywords:** bacterial cellulose, gelatin, polycaprolactone, hydroxyapatite, 3D printing, bone tissue engineering

## Abstract

Three-dimensional (3D) printing application is a promising method for bone tissue engineering. For enhanced bone tissue regeneration, it is essential to have printable composite materials with appealing properties such as construct porous, mechanical strength, thermal properties, controlled degradation rates, and the presence of bioactive materials. In this study, polycaprolactone (PCL), gelatin (GEL), bacterial cellulose (BC), and different hydroxyapatite (HA) concentrations were used to fabricate a novel PCL/GEL/BC/HA composite scaffold using 3D printing method for bone tissue engineering applications. Pore structure, mechanical, thermal, and chemical analyses were evaluated. 3D scaffolds with an ideal pore size (~300 µm) for use in bone tissue engineering were generated. The addition of both bacterial cellulose (BC) and hydroxyapatite (HA) into PCL/GEL scaffold increased cell proliferation and attachment. PCL/GEL/BC/HA composite scaffolds provide a potential for bone tissue engineering applications.

## 1. Introduction

The treatment approach to bone injury and degeneration remains a critical challenge, although autografting continues as the clinical gold standard treatment option [1,2,3]. This led to increased interest in tissue engineering and regenerative medicine, which offer a new perspective for repairing bone defects. Natural bone is a heterogeneous composite material that consists of the orderly deposition of hydroxyapatite (HA) along with the type I collagen (Col) organic matrix. According to the biomimetic concept, an ideal 3D scaffold not only actively induces bone regeneration into desired shapes but also plays a significant role to bridge in bone defects. Therefore, instead of a single-use of natural (e.g., collagen, gelatin, alginate, hyaluronic acid, and chitosan) synthetic polymers (e.g., PLGA, PLA, and polycaprolactone (PCL)) and bioceramics (the calcium phosphates (Ca/P) as hydroxyapatite (HAp), the bioactive glasses and the glass-ceramics), composite forms of them have been widely used for bone tissue engineering [4,5]. Compared with these natural materials, bacterial cellulose nanocrystal (BC) has much higher mechanical properties, which are required in most cases when used as a scaffold in bone tissue engineering There are remarkable features of bacterial cellulose that make it applicable in bone tissue engineering: biocompatibility, promoting cellular interactions and tissue development, having interconnected porous structure and significant effects on cell adhesion and proliferation, high purity level, microporosity, biodegradability, bio-absorbable, non-toxicity, resembling extracellular matrix of living tissue, and crystallinity [6,7,8,9,10].

Gelatin (Gel) is procured with denaturation of insoluble fibrous collagen protein. This denaturated material does not show resorbability or antigenicity in vivo [11]. Some properties such as low antigenicity, abundance, biocompatibility, accessible, functional groups, cost-effectiveness, and biodegradability give regenerative, and therapeutic characteristics to gelatin [12,13]. PCL is a synthetic, and semicrystalline polymer, that exhibits outstanding mechanical strength and appropriate biodegradable properties. As a result that PCL is a hydrophobic polymer whereas BC and Gel are hydrophilic, proper methods should be explored for uniformly mixing these natural and synthetic polymers. Scaffolds by a manufacture with various processing methods are not intended to be permanent implants. They will ideally facilitate host cells to deposit extracellular matrix (ECM) and replace the scaffold structure over time.

The 3D architecture of the scaffold should be highly porous with an interconnected structure to allow cell and nutrient migration. The scaffold surface should also be optimized to facilitate cell attachment, proliferation, and differentiation. Nowadays, such traditional graft processing methods such as solvent casting, particulate leaching, phase separation, lyophilization, gas foaming, and electrospinning cannot precisely control uniformity, surface geometry, pore size distribution, and interconnectivity of the scaffolds. Therefore, it is not easy for conventional techniques to create patient-specific component scaffolds with various geometrical shapes. 3D printing, as alternatively additive fabrication processes, is used for designing, and fabricating of scaffolds with designed shape, interconnected porosity, and controlled chemistry and offers an innovative material processing method to advance the manufacture artificial bone scaffolds based on the patient data and therefore the treatment of bone injuries in a clinical application [14,15,16]. Combined with appropriate techniques and innovative technologies, bone engineering uses a variety of biomaterials to mimic native bone structure. The role of biomaterials which are used in tissue engineering is to facilitate and support functions of the damaged site in the human body and to provide structure for cell specialization, migration, and population [17]. The scaffold gives structural support for cell proliferation, maintenance of cell function, and extracellular matrix (ECM) formation [18,19].

Although there are many studies concerning PCL/Gel/HA-based formulations, based on the existing knowledge, this is the unique study reporting 3D printing results of PCL/GEL/HA and BC containing PCL/GEL/HA. Incorporation of bacterial cellulose and HA with PCL/GEL was analyzed by testing tensile strength and breaking elongation to determine how mechanically affected it was. In-vitro experiments have proven the effect of bacterial cellulose and HA presence on PCL/GEL scaffold for cell viability.

In this study, PCL/GEL/BC/HA scaffolds were manufactured using 3D printing. The FTIR analysis was performed to understand the content of the materials. DSC thermogram was drawn to analyze samples thermally. The tensile testing machine was used to analyze mechanically. Cell attachment on the scaffold surfaces was visualized with optical microscopy and scanning electron microscopy (SEM). To understand the toxic effect of 3D structures on cells, an in vitro study was performed with osteoblast cells.

## 2. Materials and Methods

### 2.1. Materials

D(+)-Glucose anhydrous for biochemistry (*M*_w_ = 180.16 g/mol, C_6_H_12_O_6_), disodium hydrogen phosphate (*M*_w_ = 141.96 g/mol, Na_2_HPO_4_), acetic acid (glacial) (100% purity, *M*_w_ = 60.05 g/mol, CH_3_COOH), sodium hydroxide (*M*_w_ = 40.00 g/mol, NaOH), sulfuric acid (95–97% purity, *M*_w_ = 98.08 g/mol, H_2_SO_4_), formic acid (98–100% purity, *M*_w_ = 46.03 g/mol molecular, CH_2_O_2_), and glycerol (*M*_w_ = 92.1 g/mol, C_3_H_8_O_3_) were purchased from Merck KGaA, Darmstadt, Germany. Peptone from animal tissue from meat with suitability for cell and plant culture, citric acid (99% purity, *M*_w_ = 192.12 g/mol, C_6_H_8_O_7_), gelatin from porcine skin (powder, gel strength ~300 g Bloom, Type A, BioReagent, for electrophoresis, suitable for cell culture), polycaprolactone (PCL) with 80,000 average *M*_n_ were bought from Sigma-Aldrich, St. Louis, MO, USA. Yeast extract and agar bacteriological were purchased from Biolife, Italia. Hydroxyapatite (*M*_w_ = 502.31 g/mol) from was bought from Oerlikon Metco, Wohlen, Switzerland.

### 2.2. Methods

#### 2.2.1. Bacterial Cellulose Nanocrystals Production

S. Hestrin and M. Schramm (HS) medium was prepared in distilled water with mixing for 2.0 wt % D-glucose, 0.5 wt % peptone, 0.5 wt % yeast extract, 0.27 wt % disodium hydrogen phosphate, and 0.115 wt % citric acid, and its pH at 5.0–6.0 with acetic acid [20]. The *Glucanoacetobacter xylinus* was transposed to HS medium for the production of bacterial cellulose. The bacterial cellulose production process was carried out in static condition at ~28 °C for 10 days. At the end of 10 days, the purification process was carried out for removing cell culture and other impurities. Produced bacterial cellulose membrane was sterilized using autoclave machine at 120 °C for 20 min for obtaining completely pure, and sterile BC membrane. These oven-dried membranes were processed with 50% (*v*/*v*) sulfuric acid solution in a cellulose/acid ratio of approximately 20 g/L, at 50 °C for 2 h. Hydrolysis reactions were discontinued by diluting the reactions 8-fold with cold deionized water. To precipitate the bacterial cellulose membrane, the centrifuge was operated at 6000 rpm for 15 min at 20 °C. The bacterial cellulose membrane suspension washed with deionized water and centrifuged several times. Lastly, the suspension was dialyzed in deionized water to neutral pH, followed by freeze-drying.

#### 2.2.2. Preparation of Solutions and Production of 3D Printed Scaffolds

Firstly, PCL and Gel were blended in a mass ratio of 1:1 (*w*/*w*), and the blend was dissolved in the mixed solvent of formic acid/acetic acid (1:1 *v*/*v*) at a concentration of 14 % (*w*/*w*). BC and HA were added into PCL/Gel and PCL/Gel/BC at desired proportions given in Table 1, respectively, and then stirred at room temperature until well-dispersed nanoparticles in solution were obtained. The 3D printing process was carried out to produce four different scaffold compositions at 50%, 60%, 70%, and 80% infill rate. The 3D printed scaffolds were fabricated by using a modified 3D printer (Ultimaker 2+, Ultimaker, Geldermalsen, Netherlands), which utilized a fused deposition modeling (FDM) system. It included a computer-aided design (CAD) technology using a heatable build-plate. A digital syringe pump was connected to 3D printer to control the flow rate of solutions feeding into a syringe with an 0.5 mm nozzle diameter. 3D printed scaffolds were fabricated using conditions in build-plate temperature of 38 °C, the flow rate of 0.2 mL/h, 0.03 mm distance between needle and platform. The parameters which were used for fabrication of the scaffolds were summarized in Table 2.

#### 2.2.3. Morphological, Dimensional and Chemical Analysis of 3D Printed Scaffolds

Dimensional and morphological analyzes of the 3D printed scaffolds were characterized using an optical microscope (BX51M model, Olympus, Tokyo, Japan) and scanning electron microscopy (SEM, EVO MA-10, Zeiss, Oberkochen, Germany). The scaffold samples which were analyzed using SEM were coated with gold using a sputter coater (SC 7620 model, Quorum Technologies, East Sussex, UK) for 90 s, 18 mA, and observed by SEM with an accelerating voltage of 10 kV. Pore size diameters were measured from randomly selected 105 pores from SEM images (taken at 75× magnification) with Image J software. The existence of chemical groups or bonds was analyzed using Fourier Transform Infrared Spectroscopy (FT/IR-4700, Jasco, Tokyo, Japan). The wavelength value of the FTIR device ranges between 400 and 4000 cm^−1^.

#### 2.2.4. Mechanical and Thermal Analysis of 3D Printed Scaffolds

The mechanical properties of the scaffolds were conducted at room temperature on a tensile testing machine (EZ-LX model, Shimadzu, Kyoto, Japan) with a load cell of 10 N and crosshead speed of 5 mm/min. Samples were prepared rectangular strips of 10 mm × 5 mm, and the thickness of the samples was measured with a digital caliper (3 mm). Thermal properties of the scaffold were analyzed by using differential scanning calorimeter (DSC) (DSC-60 Plus model, Shimadzu, Kyoto, Japan) in a temperature range of 25–550 °C at a heating rate of 10 °C/min.

#### 2.2.5. Cell Culture Test for 3D Printed Scaffolds

The 3D printed scaffolds with round pieces (~2.1 mm in thickness and discs of 6 mm in diameter) were transferred to 96-well plates and sterilized under ultraviolet (UV) light overnight. Then samples were incubated in cell culture medium for 2 h before cell seeding. Human osteoblast cells (ATCC) suspension (1 × 10^4^ cells/well in Dulbecco’s modified eagle’s medium (DMEM) supplemented with 10% fetal bovine serum (FBS) and 1% penicillin/streptomycin) were seeded onto scaffold for specific intervals at 37 °C with 5% CO_2_ followed by the examination of cell adhesion and cell viability.

A 3-(4,5-dimethylthiazol-2-yl)-2,5-diphenyl tetrazolium bromide (MTT) assay was performed to analyze cell viability after 1, 3, and 7 days of incubation periods on 3D printed scaffolds. On the day of the test, the samples were washed with cold PBS (pH 7.4), followed by addition of MTT solution at the concentration of 0.5 mg/mL (0.5 mL) and incubated for 5 h at 37 °C with 5% CO_2_. According to the manufacturer’s protocol, the supernatant was then removed gently followed by the addition of 1.5 mL of dimethylsulfoxide (DMSO). Plates were again incubated for 15 min at 37 °C with 5% CO_2_, and the absorbance was measured using a microplate reader in 560 nm wavelength.

Morphological analyzes of the 3D printed scaffolds were characterized using a confocal microscope (Zeiss Model LSM700, Zeiss, Oberkochen, Germany) and scanning electron microscopy (SEM, EVO MA-10, Zeiss, Oberkochen, Germany). After 7 days of incubation, samples were fixed with 2.5% glutaraldehyde (Sigma, St. Louis, MO, USA) for 2 h and then dehydrated through serial dilutions of ethanol (30%, 50%, 70%, 90%, and 99%). Dried samples which were analyzed using SEM were sputter-coated with gold (SC 7620 model, Quorum Technologies, Sacramento, CA, USA) for 90 s and observed by SEM (EVO MA-10, Zeiss, Oberkochen, Germany) with an accelerating voltage of 10 kV.

After 7 days of incubation, the 3D printed scaffolds fixed with 4% formaldehyde for 1 h followed by washing using PBS. The samples were permeabilized in 0.1% Triton X-100 in PBS for 30 min, followed by washing with PBS. The samples were then stained with DAPI (1 µg/mL) for 40 min at room temperature and again washed with PBS. The cell fluorescence was determined using a fluorescence microscope with a 20×.

## 3. Results and Discussion

**Figure 1:**
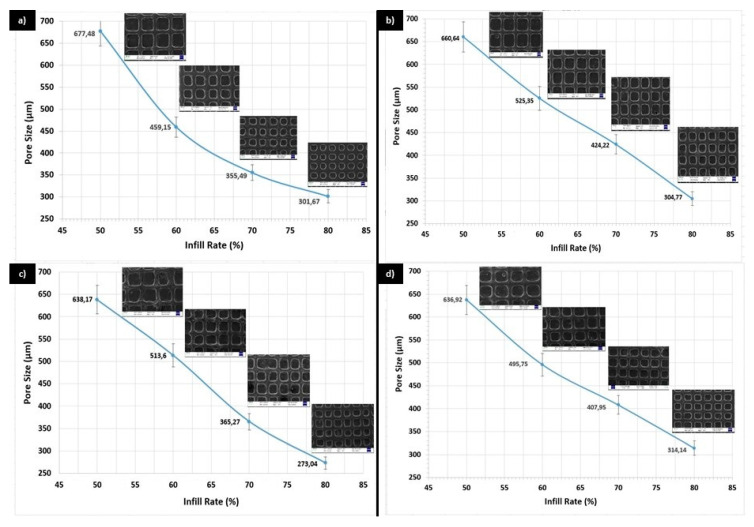
Mean pore size for (**a**) PCL/GEL, (**b**) PCL/GEL/BC, (**c**) PCL/GEL/BC/0.25%HA, (**d**) PCL/GEL/BC/0.50%HA solution at 50%, 60%, 70%, and 80% infill rate, respectively.

**Figure 2:**
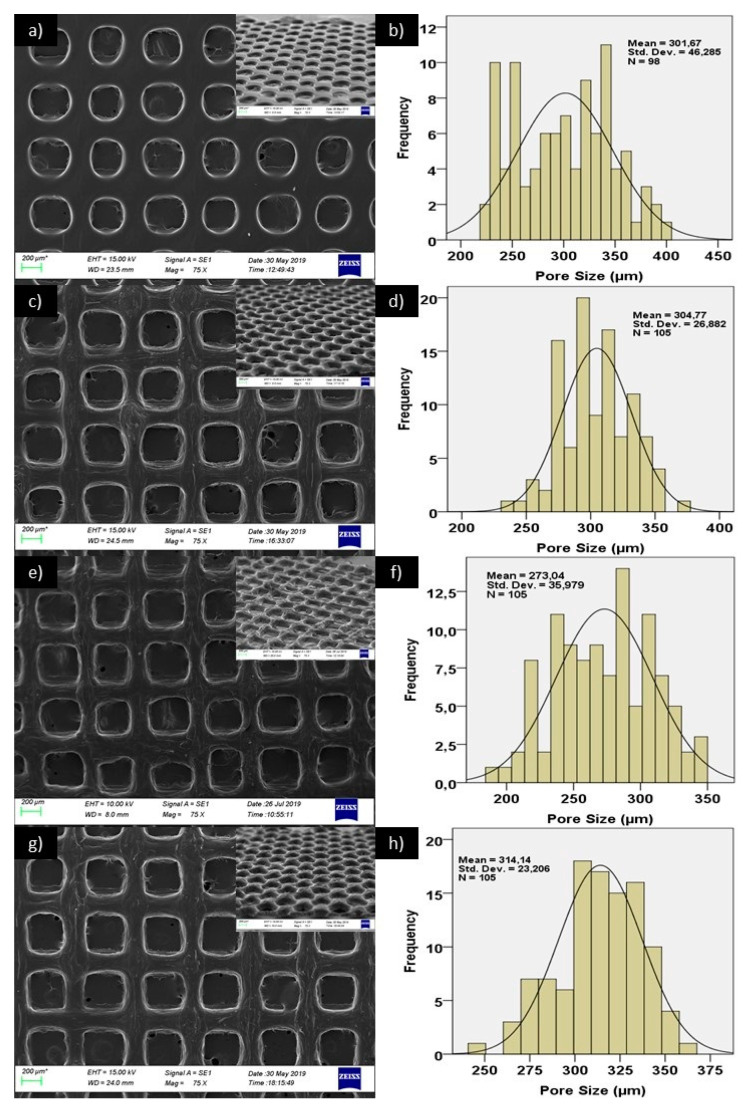

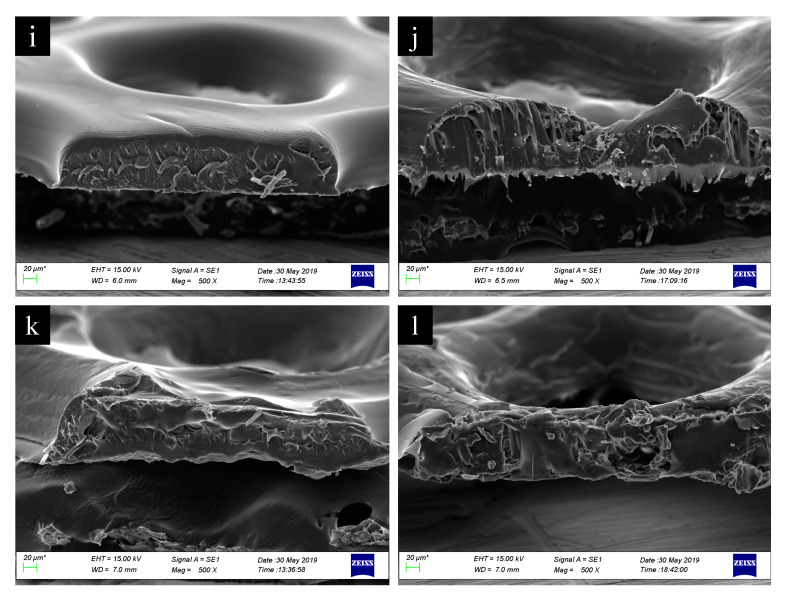
SEM images of (**a**,**i**) PCL/GEL, (**c**,**j**) PCL/GEL/BC, (**e**,**k**) PCL/GEL/BC/0.25%HA, (**g**,**l**) PCL/GEL/BC/0.50%HA scaffolds and pore size histiograms of (**b**) PCL/GEL, (**d**) PCL/GEL/BC, (**f**) PCL/GEL/BC/0.25%HA and **h**) PCL/GEL/BC/0.50%HA scaffolds.

The mean pore sizes for the printed scaffolds with four different infill rate were shown in Figure 1a for PCL/GEL, Figure 1b for PCL/GEL/BC, Figure 1c for PCL/GEL/BC/0.25%HA, and Figure 1d for PCL/GEL/BC/0.50%HA. The pore size is an essential parameter for scaffold, which is fabricated for BTE. This parameter affects the characteristics and quantity of new tissue [21]. Ideal pore size is not well defined for BTE scaffolds. For osteoconduction, pore sizes (100–400 µm) are recommended as optimal. Pore sizes (>300 µm) are proposed to increase bone formation by way of vascularization [22,23]. Pore sizes (<100 µm) cannot be qualified from the point of cell migration and mass transport and can cause the formation of endochondral cartilage. Larger pore sizes can result in redundant void space and affect adversely mechanical properties of BTE scaffold. These are sufficient for capillary formation. Therefore, the pore size for BTE scaffold should be small enough to procure mechanical integrity, but large enough to carry out the waste and nutrient diffusion requirement of bone tissue [21,23]. Infill rate refers to the percentage of infill. In the Simplify program, the fill rate is used to give the pores to the solid model. If the infill rate is selected as 100%, a completely solid structure with no pores is formed. If certain values are infilled, the program divides the previously drawn completely non-porous solid model into certain parts and forms a porous structure. As shown in Figure 1, the pore size decreased in relation to the filling ratio. According to the results, optimal pore sizes were obtained at 80% infill rate in this study. Figure 2 shows SEM images and pore size distribution of the 3D printed scaffolds at 80% infill rate. The structural integrity and the porosity of the fabricated 3D scaffolds were displayed in this figure. As shown in Figure 2, due to the presence of BC and HA nanoparticles, surface morphology has changed from smooth rough to between interconnected pores. It can be seen that while the pore structures in the 3D printed PCL/Gel scaffold were a round shape, they took the rectangular form with the addition of BC and HA. The mean pore size increased from 301.67 ± 46.3 µm to 304.77 ± 26.9 µm by the addition of BC in PCL/Gel. A similar trend was observed by addition of 0.50 wt % HA in PCL/Gel/BC, that is, it increased to 314.14 ± 23.2 µm. In addition, the mean pore size decreased with the presence of 0.25 wt % HA in PCL/Gel/BC, while heterogeneous pore size distribution was observed at this concentration when compared to the those in 0.50 wt % HA.

**Figure 3:**
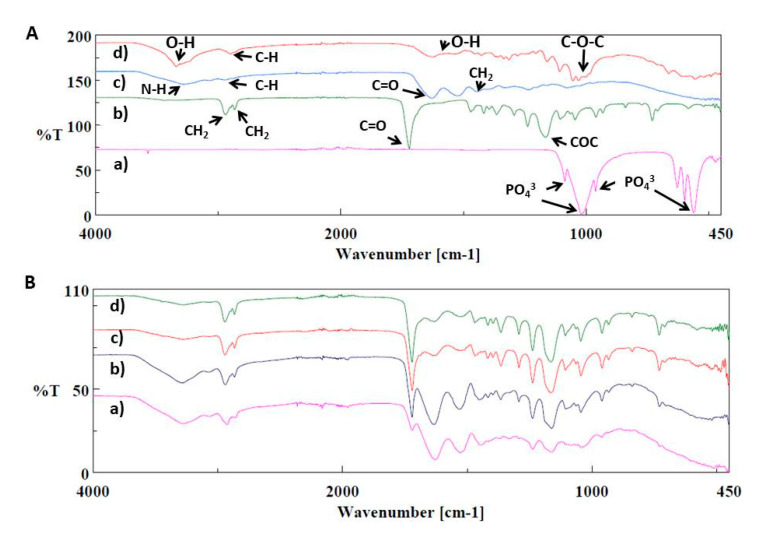
FTIR spectrums of (**A**,**a**) pure hydroxyapatite, (**A**,**b**) pure PCL, (**A**,**c**) pure gelatin, (**A**,**d**) pure bacterial cellulose, (**B**,**a**) PCL/GEL, (**B**,**b**) PCL/GEL/BC, (**B**,**c**) PCL/GEL/BC/0.25%HA, (**B**,**d**) PCL/GEL/BC/0.50%HA.

The functional groups related to pure polymers and their composites were identified by FTIR spectroscopy. In Figure 3A, the FTIR spectrum of pure polymers and synthesized bacterial cellulose is given with their functional groups. In Figure 3A(a), hydroxyapatite (HA) had main absorption peaks at ~1087.7 cm^−1^ (PO_4_^3−^, asymmetric stretching), ~1018.2 cm^−1^ (PO_4_^3−^, asymmetric stretching), ~962.3 cm^−1^ (PO_4_^3−^, symmetric stretching), ~628.7 cm^−1^ (OH bending), ~597.8 cm^−1^, and ~561.2 cm^−1^ is due to the asymmetric bend of PO_4_^3−^ [24,25]. The spectrum of PCL (Figure 3A(b)) showed several peaks around ~2940.9 cm^−1^, ~2865 cm^−1^, ~1720.2 cm^−1^, ~1293 cm^−1^, ~1238.1 cm^−1^, ~1164 cm^−1^ which were associated with asymmetric CH_2_ stretching, symmetric CH_2_ stretching, carbonyl stretching (C=O), again asymmetric COC stretching, asymmetric COC stretching, symmetric COC stretching, respectively [26]. Gelatin showed various bands at ~3277.4 cm^−1^ which corresponded the N–H stretch coupled with hydrogen bonding. The peak at ~2935.1 cm^−1^ is due to the alkenyl C–H stretching. C=O stretching was observed at ~1628.6 cm^−1^. Other peaks were found at ~1443.5 cm^−1^ (CH_2_), ~1234.2 cm^−1^ (NH), ~1079 (C–O stretching) (Figure 3 A(c)) [27]. The FTIR spectrum of the BC was given in Figure 3A(d). There were detected broad absorption peaks for BC some of them are: ~3342 cm^−1^ (OH stretching), ~2896.6 cm^−1^ (CH stretching), ~1622.8 cm^−1^ (related to the bending mode of the naturally absorbed water), ~14297.1 cm^−1^ (CH_2_ and COH bending inside of plane), ~664.4 cm^−1^ (C–OH bending out of plane) [28]. Figure 3B showed the FTIR spectrums of the composites. In Figure 3B(a), there were observed some shifts after the interaction between the PCL and GEL such as the peak for pure PCL at 2940.9 cm^−1^ was shifted to 2923.6 cm^−1^, another peak for pure GEL which is observed at 1519.6 cm^−1^ was shifted to 1526.4 cm^−1^. The absorption peak for pure GEL at 1332.6 cm^−1^ was also observed at PCL/GEL composite. There was found another difference that the peaks at 1079 cm^−1^ (pure GEL) and 1045 cm^−1^ (pure PCL), these peaks gave mean value at ~1044.3 cm^−1^ which can be evidence of the interaction between the Gelatin and PCL polymers. By the addition of 0.25%BC into PCL/GEL, BC peaks observed in the mixture at ~2865.7 and ~1530.2 cm^−1^ points and some shifts were observed after the interaction (Figure 3B(b)). In Figure 3B(c)*,* the FTIR spectrum of PCL/GEL/BC/0.25%HA is shown. The specific GEL absorption peaks were observed at ~3278.4 and ~2942.8 cm^−1^ in the PCL/GEL/BC/0.25%HA spectrum. In addition, by the addition of 0.25%HA, there were observed small shifts between the PCL/GEL/BC and PCL/GEL/BC/0.25%HA composites. With the addition of 0.50%HA into the PCL/GEL/BC composite, there was not observed the sharp difference between the spectrums. The peaks which were observed for the PCL/GEL/BC/0.25%HA composite were seen in the spectrum of PCL/GEL/BC/0.50%HA composite (Figure 3B(d)).

**Figure 4:**
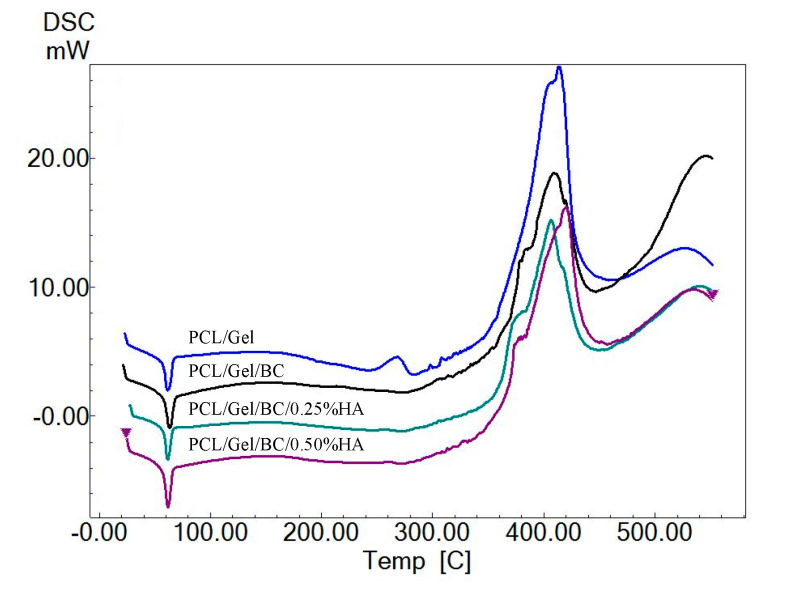
DSC thermogram of PCL/GEL, PCL/GEL/BC, PCL/GEL/BC/0.25%HA, and PCL/GEL/BC/0.50%HA scaffolds.

DSC thermograms were shown for PCL/GEL, PCL/GEL/BC, PCL/GEL/BC/0.25%HA, and PCL/GEL/BC/0.50%HA in Figure 4. PCL has a melting temperature at approximate 58–60 °C. The decomposition temperature (*T*_d_) of PCL is 350 °C [29]. As shown in Figure 4, four endothermic peaks at approximate 60 °C observed which are related to melting temperature (*T*_m_) of PCL [30]. It can be observed that the scaffolds showed a degradation step in the range of 387–430 °C. The maximum decomposition rate of 420 °C was observed for PCL/GEL/BC/0.50%HA. These conclusions agree with the values which are reported in the literature [31,32,33]. In addition, endothermic peaks (*T*_m_) at 61.60, 61.70, 62.04 and 63.54 °C were observed which are related to melting temperature of gelatin [34].

To investigate the structural integrity of the PCL/GEL, PCL/GEL/BC, PCL/GEL/BC/0.25%HA, and PCL/GEL/BC/0.50%HA composites as bone scaffolds, their mechanical properties determined using the tensile testing device. Tensile testing results in Table 3 showed that PCL/GEL had both the highest tensile strength (6.61 MPa) and the highest elongation at break (4.66%) values which point out good deformability and flexibility [35]. By the addition of 0.25 wt % BC into the PCL/GEL matrix, tensile strength value decreased, but strain at break increased to the value of 5.37%. As a result of this situation, it could be said that the blending of BC with PCL/GEL did not increase the strength value. This might be due to the weak physical interactions between the chains of blending polymers under loading [35]. As the 0.25 wt % HA added to the PCL/GEL/BC composite, tensile strength and elongation values decreased. By the addition of the 0.50 wt % HA, tensile strength and strain at break values increased again but still lower than PCL/GEL and PCL/GEL/BC composites. It could be said that composites produced with high HA (0.50 wt %) exhibited higher strength and strain values than those produced with low HA (0.25%) [36]. The balance between the bonding and the deposition is an efficient way to fabricate composite scaffolds and it may influence the mechanical properties of the 3D printed composite scaffolds [37]. Contrary to expectations, the addition of HA did not improve the mechanical properties. One of the main reasons behind poor strength was the imperfect bonding between layers at z-axis. During the printing process, the layers did not fuse properly. Hence, the tensile behaviors may also be affected by the defects caused by the printing path.

**Figure 5:**
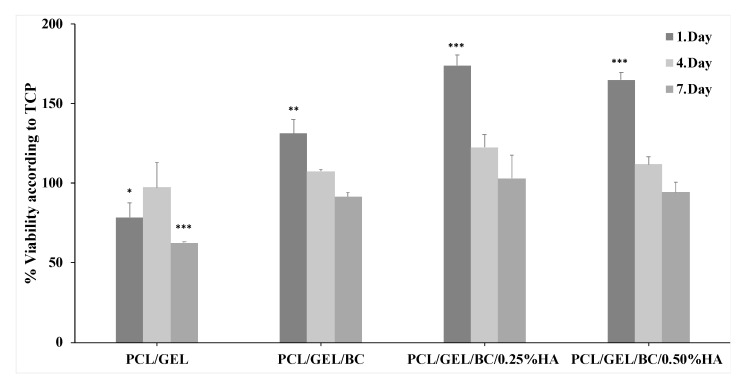
Cell viability analysis of PCL/GEL, PCL/GEL/BC, PCL/GEL/BC/0.25%HA, and PCL/GEL/BC/0.50%HA scaffolds. Data are the means ± S.E.; *n* = 3; * *p* ≤ 0.05, ** *p* ≤ 0.01, *** *p* ≤ 0.001 versus TCP, ANOVA, Tukey–Kramer multiple comparison test.

Tissue engineering scaffolds and their degradation products should not be toxic to the cells. Therefore, in vitro biological properties of tissue culture plate (TCP) and 3D printed PCL/GEL, PCL/GEL/BC/0.25%HA and PCL/GEL/BC/0.50%HA scaffolds were examined by cell viability and cell-scaffold interaction. The result showed that the proliferation rate of human osteoblast cells significantly increased in all 3D printed scaffolds compared to TCP after 1 day of incubation (Figure 5). The presence of BC into PCL/GEL scaffold increased cell viability.

It is clearly shown that in the case of scaffolds containing HA, the cell viability was found to be significantly increased irrespective of their HA content when compared to PCL/GEL and PCL/GEL/BC scaffolds. Furthermore, the addition of 0.25 wt % HA to PCL/GEL/BC showed excellent cell viability during the 3 and 7 days of the incubation period.

**Figure 6:**
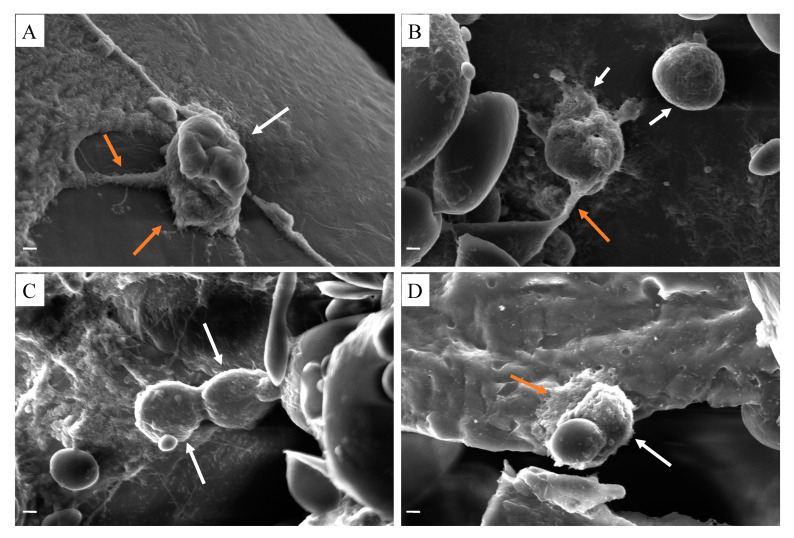
SEM images of human osteoblast cells (white arrow) and pseudopodia-like extended morphology (orange arrow) on (**A**) PCL/GEL, (**B**) PCL/GEL/BC, (**C**) PCL/GEL/BC/0.25%HA, (**D**) PCL/GEL/BC/0.50%HA scaffolds (scale bar 2 µm).

**Figure 7:**
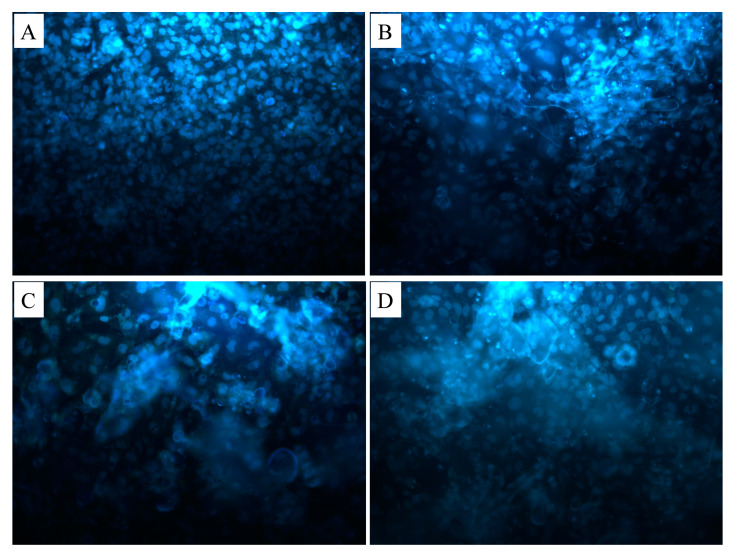
Fluorescence microscopy images of human osteoblast cells on (**A**) PCL/GEL, (**B**) PCL/GEL/BC, (**C**) PCL/GEL/BC/0.25%HA, (**D**) PCL/GEL/BC/0.50%HA scaffolds.

Cell morphology on the 3D printed scaffolds was shown in Figure 6. Osteoblast cells were seeded on scaffolds. After 7 days of cell culture, the morphological structures of cells adhesion to PCL/GEL (Figure 6A), PCL/GEL/BC (Figure 6B), PCL/GEL/BC/0.25%HA (Figure 6C), and PCL/GEL/BC/0.50%HA (Figure 6D) scaffolds were demonstrated by SEM analysis. The results clearly showed that osteoblast cells tend to proliferate and adapt to the extracellular matrix on which they were cultured on the scaffold. The printed composite scaffolds showed a more rounded and interconnected morphology. The result clearly showed that culturing cells on PCL/Gel composite scaffold exhibited a spread shape morphologies with a pseudopodia-like extended structures to the scaffolds. On 3D printed scaffolds, osteoblast cells displayed extensive cytoplasmic dendritic structures (Figure 6A,B,D), similar to a characteristic morphology of osteocytes. Cell adhesion on the 3D printed scaffolds was observed using fluorescent staining with DAPI after 7 days of cell seeding (Figure 7). It was also recognized that the osteoblast cells had an excellent cell attachment on the 3D printed scaffolds.

## 4. Conclusions

In this study, porous scaffolds were fabricated using 3D printing for four different composites at four different infill rates from fifty to eighty percent. It was aimed to produce scaffolds with ideal/optimal pore size and high uniformity ratio. All three-dimensional printed scaffolds which have 80% infill rate both have an ideal pore size for bone tissue engineering and have a uniformity ratio of more than 90%. The composite’s tensile strength, although PCL/Gel/BC blends increased compared to that of PCL/Gel/BC/HA blends, was considerably lower than that of the PCL/Gel. The PCL/GEL/BC/0.25%HA scaffold demonstrated good cell viability and cell adhesion when compared to PCL/GEL, PCL/GEL/BC and PCL/GEL/BC/0.50%HA. Altogether, these results suggest that the prepared 3D composite scaffolds have a great potential to develop better bone implants for biomedical applications.

## Figures and Tables

**Table 1:** Concentrations of polycaprolactone (PCL)/gelatin (GEL)/bacterial cellulose (BC)/ hydroxyapatite (HA) solutions.

Solutions	PCL Conc. (wt %)	GEL Conc. (wt %)	BC Conc. (wt %)	HA Conc. (wt %)
PCL/GEL	14	14	-	-
PCL/GEL/BC	14	14	0.25	-
PCL/GEL/BC/0.25%HA	14	14	0.25	0.25
PCL/GEL/BC/0.50%HA	14	14	0.25	0.50

**Table 2:** Process parameters for 3D printing of scaffolds.

Parameters	Values
Number of Layers	10
Flow Rate (mL/h)	0.2
Needle Tip Outer Diameter (mm)	0.5
Platform Temperature (°C)	38
Room Temperature (°C)	25
Infill Rate (%)	50, 60, 70, and 80

**Table 3:** Tensile Testing Results for PCL/GEL, PCL/GEL/BC, PCL/GEL/BC/0.25%HA, and PCL/GEL/BC/0.50%HA.

Samples	Tensile Strength (MPa)	Strain at Break (%)
PCL/GEL	6.61 ± 1.99	4.66 ± 1.75
PCL/GEL/BC	2.17 ± 0.21	5.37 ± 2.33
PCL/GEL/BC/0.25%HA	1.08 ± 0.34	3.29 ± 1.41
PCL/GEL/BC/0.50%HA	1.58 ± 0.19	3.97 ± 2.27

## References

[1] Langer R. (2000). Biomaterials in drug delivery and tissue engineering: One laboratory’s experience. Acc. Chem. Res..

[2] Bonassar L.J., Vacanti C.A. (1998). Tissue engineering: The first decade and beyond. J. Cell Biochem..

[3] Atala A. (2019). Tissue engineering and regenerative medicine. Rejuvenation Res..

[4] Ivkovic A., Marijanovic I., Hudetz D., Porter R. (2011). Regenerative medicine and tissue engineering in orthopaedic surgery. Regen. Orthop..

[5] Surmenev R.A., Shkarina S., Syromotina D.S., Melnik E.V., Shkarin R., Selezneva I.I., Ermakov A.M., Ivlev S.I., Cecilia A., Weinhardt V. (2019). Characterization of biomimetic silicate- and strontium-containing hydroxyapatite microparticles embedded in biodegradable electrospun polycaprolactone sca ff olds for bone regeneration. Eur. Polym. J..

[6] Noh Y.K., Costa A.D.S.D., Park Y.S., Du P., Kim I.-H., Park K. (2019). Fabrication of bacterial cellulose-collagen composite scaffolds and their osteogenic effect on human mesenchymal stem cells. Carbohydr. Polym..

[7] Zaborowska M., Bodin A., Bäckdahl H., Popp J., Goldstein A., Gatenholm P. (2010). Microporous bacterial cellulose as a potential scaffold for bone regeneration. Acta Biomater..

[8] Ramani D., Sastry T.P. (2014). Bacterial cellulose-reinforced hydroxyapatite functionalized graphene oxide: A potential osteoinductive composite. Cellulose.

[9] Abdelraof M., Hasanin M.S., Farag M.M., Ahmed H.Y. (2019). Green synthesis of bacterial cellulose/bioactive glass nanocomposites: Effect of glass nanoparticles on cellulose yield, biocompatibility and antimicrobial activity. Int. J. Biol. Macromol..

[10] Huang Y., Wang J., Yang F., Shao Y., Zhang X., Dai K. (2017). Modification and evaluation of micro-nano structured porous bacterial cellulose scaffold for bone tissue engineering. Mater. Sci. Eng. C.

[11] Yin Y., Ye F., Cui J., Zhang F., Li X., Yao K. (2003). Preparation and characterization of macroporous chitosan–gelatin/β-tricalcium phosphate composite scaffolds for bone tissue engineering. J. Biomed. Mater. Res..

[12] Su K., Wang C. (2015). Recent advances in the use of gelatin in biomedical research. Biotechnol. Lett..

[13] Song J.-H., Kim H.-E., Kim H.-W. (2008). Production of electrospun gelatin nanofiber by water-based co-solvent approach. J. Mater. Sci. Mater. Med..

[14] Ma H., Feng C., Chang J., Wu C. (2018). 3D-printed bioceramic scaffolds: From bone tissue engineering to tumor therapy. Acta Biomater..

[15] Zhang L., Yang G., Johnson B.N., Jia X. (2019). Three-dimensional (3D) printed scaffold and material selection for bone repair. Acta Biomater..

[16] Turnbull G., Clarke J., Picard F., Riches P., Jia L., Han F., Li B., Shu W. (2018). 3D bioactive composite scaffolds for bone tissue engineering. Bioact. Mater..

[17] Shrivats A.R., Mcdermott M.C., Hollinger J.O. (2014). Bone tissue engineering: State of the union. Drug Discov. Today.

[18] Paula A., Madrid M., Paola A., Vrech S.M., Sanchez M.A. (2019). Advances in additive manufacturing for bone tissue engineering scaffolds. Mater. Sci. Eng. C.

[19] Hutmacher D.W. (2000). Scaffolds in tissue engineering bone and cartilage. Biomaterials.

[20] Hestrin S., Schramm M. (1953). Synthesis of cellulose by Acetobacter xylinum: 2. Preparation of freeze-dried cells capable of polymerizing glucose to cellulose. Biochem. J..

[21] Karageorgiou V., Kaplan D. (2005). Porosity of 3D biomaterial scaffolds and osteogenesis. Biomaterials.

[22] Cyster L.A., Grant D.M., Howdle S.M., Rose F.R.A.J., Irvine D.J., Freeman D., Scotchford C.A., Shakesheff K.M. (2005). The influence of dispersant concentration on the pore morphology of hydroxyapatite ceramics for bone tissue engineering. Biomaterials.

[23] Roosa S.M.M., Kemppainen J.M., Moffitt E.N., Krebsbach P.H., Hollister S.J. (2010). The pore size of polycaprolactone scaffolds has limited influence on bone regeneration in an in vivo model. J. Biomed. Mater. Res. Part. A.

[24] Andrea P., Sossa F., Giraldo B.S., Clemencia B., Garcia G., Parra E.R., Jose P., Arango A. (2018). Comparative study between natural and synthetic Hydroxyapatite: Structural, morphological and bioactivity properties. Rev. Matér..

[25] Koutsopoulos S. (2002). Synthesis and characterization of hydroxyapatite crystals: A review study on the analytical methods. J. Biomed. Mater. Res..

[26] Elzein T., Nasser-eddine M., Delaite C., Bistac S., Dumas P. (2004). FTIR study of polycaprolactone chain organization at interfaces. J. Colloid Interface Sci..

[27] Premlatha T.S., Kothai S. (2015). Synthesis, Characterization and Antibacterial Activity of Gelatin-Herb Nanocomposite. Asian J. Biomed. Pharm. Sci..

[28] Auta R., Adamus G., Kwiecien M., Radecka I., Hooley P. (2017). Production and characterization of bacterial cellulose before and after enzymatic hydrolysis. Afr. J. Biotechnol..

[29] Wang F., Shor L., Darling A., Khalil S., Sun W., Güçeri S., Lau A. (2004). Precision extruding deposition and characterization of cellular polycaprolactone tissue scaffolds. Rapid Prototyp. J..

[30] Ghorab D., Amin M., Khowessah O. (2011). Colon-targeted celecoxib-loaded Eudragit S100-coated polycaprolactone microparticles: Preparation, characterization and in vivo evaluation in rats. Drug Deliv..

[31] Lozano-Sánchez L., Bagudanch I., Sustaita A.O., Iturbe-Ek J., Elizalde L.E., Garcia-Romeu L.M., Elías-Zúñiga A. (2018). Single-Point Incremental Forming of Two Biocompatible Polymers: An Insight into Their Thermal and Structural Properties. Polymers.

[32] Cervantes A.L., Lopez I.D., Sanchez J.D.O.B., Garcia A.L.G. (2013). Effects of surface texturing on the performance of biocompatible UHMWPE as a bearing material during in vitro lubricated sliding/rolling motion. J. Mech. Behav. Biomed. Mater..

[33] Khan R.A., Parsons A.J., Jones I.A., Walker G.S., Rudd C.D. (2010). Preparation and Characterization of Phosphate Glass Fibers and Fabrication of Poly (caprolactone) Matrix Resorbable Composites. J. Reinf. Plast. Compos..

[34] Asma C., Meriem E., Mahmoud B., Djaafer B. (2014). Physicochemical Characterization of Gelatin-GMC Composite Edibles Films from Polyion-complex Hydrogels. J. Chil. Chem. Soc..

[35] Zhang Y., Ouyang H., Chwee T.L., Ramakrishna S., Huang Z.M. (2005). Electrospinning of gelatin fibers and gelatin/PCL composite fibrous scaffolds. J. Biomed. Mater. Res..

[36] Kim J.W., Shin K.H., Koh Y.H., Hah M.J., Moon J., Kim H.E. (2017). Production of poly(ε-caprolactone)/hydroxyapatite composite scaffolds with a tailored macro/micro-porous structure, high mechanical properties, and excellent bioactivity. Materials.

[37] Zhou Y.G., Zou J.R., Wu H.H., Xu B.P. (2020). Balance between bonding and deposition during fused deposition modeling of polycarbonate and acrylonitrile-butadiene-styrene composites. Polym. Compos..

